# An Improved Endoscopic Automatic Classification Model for Gastroesophageal Reflux Disease Using Deep Learning Integrated Machine Learning

**DOI:** 10.3390/diagnostics12112827

**Published:** 2022-11-17

**Authors:** Hsu-Heng Yen, Hui-Yu Tsai, Chi-Chih Wang, Ming-Chang Tsai, Ming-Hseng Tseng

**Affiliations:** 1 Division of Gastroenterology, Changhua Christian Hospital, Changhua 500, Taiwan; 2 Artificial Intelligence Development Center, Changhua Christian Hospital, Changhua 500, Taiwan; 3 Department of Post-Baccalaureate Medicine, College of Medicine, National Chung Hsing University, Taichung 400, Taiwan; 4 Department of Medical Informatics, Chung Shan Medical University, Taichung 402, Taiwan; 5 Institute of Medicine, Chung Shan Medical University, Taichung 402, Taiwan; 6 Division of Gastroenterology and Hepatology, Department of Internal Medicine, Chung Shan Medical University Hospital, Taichung 402, Taiwan; 7 Information Technology Office, Chung Shan Medical University Hospital, Taichung 402, Taiwan

**Keywords:** gastroesophageal reflux disease, deep learning, transfer learning, machine learning, healthcare

## Abstract

Gastroesophageal reflux disease (GERD) is a common digestive tract disease, and most physicians use the Los Angeles classification and diagnose the severity of the disease to provide appropriate treatment. With the advancement of artificial intelligence, deep learning models have been used successfully to help physicians with clinical diagnosis. This study combines deep learning and machine learning techniques and proposes a two-stage process for endoscopic classification in GERD, including transfer learning techniques applied to the target dataset to extract more precise image features and machine learning algorithms to build the best classification model. The experimental results demonstrate that the performance of the GerdNet-RF model proposed in this work is better than that of previous studies. Test accuracy can be improved from 78.8% ± 8.5% to 92.5% ± 2.1%. By enhancing the automated diagnostic capabilities of AI models, patient health care will be more assured.

## 1. Introduction

Gastroesophageal reflux is a common physiological phenomenon, and most people may have experienced different severity of reflux [1]. However, if the frequency of gastroesophageal reflux is too high and continues, frequent symptoms or related complications, such as changes in the esophageal mucosa, are called gastroesophageal reflux disease (GERD) [2,3,4,5]. GERD is a relatively common digestive tract disease; it can cause discomfort for the patient and damage the esophageal mucosal tissue. Although GERD is further divided into erosive esophagitis and non-erosive esophagitis, most clinicians use the Los Angeles (LA) classification of esophagitis and diagnose the severity of the disease to provide appropriate treatment.

With the advancement of artificial intelligence (AI), the computer-aided diagnosis technique has become increasingly mature [6]. There is much literature in medicine that uses deep learning models to help physicians diagnose [7,8,9,10,11,12,13,14,15,16,17]. In the classification of GERD, in 2010, Pace et al. proposed a QUID questionnaire (Questionario Italiano Diagnostico) [18] and used an artificial neural network (ANN) to predict. The results of the study showed that the combination of ANN and QUID questionnaires could help distinguish whether a patient suffers from GERD or not. Huang et al. [19] proposed a hierarchical heterogeneous descriptor fusion support vector machine (HHDF-SVM) method for the diagnosis of GERD from conventional endoscopic images. In 2021, Wang et al. proposed the Gerd-VGGNet architecture [20]. This model is a deep convolutional neural network architecture with high generalization. It uses traditional RGB endoscopic images and narrow band imaging (NBI) images for analysis and uses the LA classification to predict the classification of diseases. The experiment compares four pre-trained models. The results show that selecting VGG16 as the pre-trained model has better model accuracy.

In recent years, automatic image feature extraction using pre-trained models of deep learning methods has become a modern technique [21]. In contrast to the pre-training model based on Imagenet [22], studies by Fan et al. [23] and Chang et al. [24] both confirmed that more precise image features can be extracted after fine-tuning the pre-trained network parameters on the target dataset. Yadav et al. [25] compared performance differences between machine learning and deep learning methods for facial acne binary classification using only 120 images and found that a CNN model with the LeakyReLU activation function is better than the SVM model in accuracy. It is important to note that they did not extract image features using a pre-trained model before performing classification using the SVM model.

The purpose of this study is to facilitate the endoscopist in distinguishing the LA classification in the endoscopic mucosa characters at the esophageocardiac junction during the esophagogastroduodenoscopy exam. This relevant AI assistant mode can improve the diagnosis of endoscopic classification of GERD in inexperienced endoscopists in our previous study [20]. This study attempts to improve the accuracy of the prediction system through different deep learning and machine learning techniques to assist in endoscopy of gastroesophageal reflux disease in clinical practice.

The research contributions of this paper are as follows. In this work, a two-stage process for the classification of three-class endoscopic in GERD is proposed that combines deep learning and machine learning techniques. This process includes applying transfer learning to the target data set to extract more precise image features and using machine learning algorithms to create the best classification model. As an image feature extractor, we suggest using the Gerd-VGGNet model. The extracted feature set is then classified using a random forest (RF) classifier with parameter optimization. The experimental results confirm that the prediction performance of this study is superior to that of previous studies.

## 2. Methods

### 2.1. GERD Dataset

This study aims to improve the predictive performance of Gerd-VGGNet [20] for GERD, so the same dataset is used for subsequent analysis and comparison. In this study, the NBI endoscopic image data set collected by Wang et al. [20] is used to perform a three-class classification of GERD due to previous references that showed better reproducibility in the classification of esophagitis [26,27] and better differentiation of erosive esophagitis from non-erosive reflux disease [28,29] comparing NBI with conventional images.

Images were obtained from the Chung Shan Medical University Hospital endoscopy system and image data from 496 patients were collected [20]. After processing, a total of 671 images were obtained as the development set, and the other 32 images were used as the test set. In the development set, 244 NBI endoscopic images of grades A–B, 229 images of grades C–D, and 198 normal endoscopic images were used. Furthermore, the distribution of the test set was 12 images from grades A–B, 10 images from grades C–D, and 10 normal endoscopic images.

### 2.2. Feature Extraction

Compared to traditional machine learning methods that require manual feature extraction, deep learning pre-trained models can automatically extract features from many images. To compare the effects of different deep learning pre-trained models on the classification of GERD, this study compared five image feature extraction techniques described below, where the input size of the image is 96 × 96 × 3, same as Wang et al. [20].

**Figure 1:**
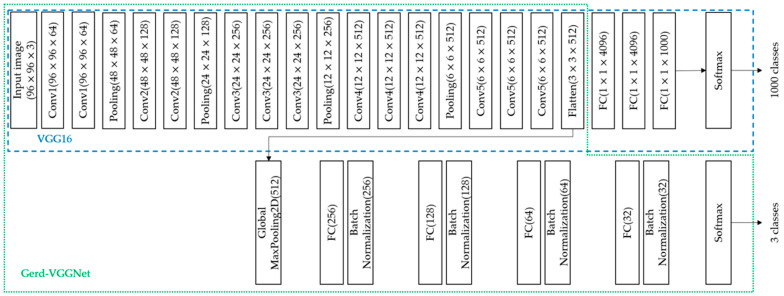
Gerd-VGGNet architecture.

(1) EfficientNetB7: The deep network architecture proposed by Tan et al. in 2019 [30]. Seven layers were used as the backbone, including the input layer, rescaling, normalization, zero padding, convolution layer, batch normalization, and activation, and then the seven blocks were connected. Each block was connected to a different sub-block. There were 813 layers in total. Our study uses this deep learning model to automatically extract 2560 image features from a global maximum pooling (GMP) layer.
(2) ResNet50: Residual learning framework proposed by He et al. in 2016 [31] to simplify the training of deeper networks than previously used. On the ImageNet dataset, residual networks with a depth of up to 152 layers were evaluated, which was eight times deeper than the VGG network, but still had lower complexity. This study uses this deep learning model to automatically extract 2048 image features from a GMP layer.
(3) InceptionResNetV2: Proposed by Szegedy et al. in 2017 [32], InceptionResNetV2 combined the Inception Module and the residual network. Better model accuracy was obtained by deepening the network and using asymmetric convolutional layers and 1 × 1 convolutional layers to implement the model. This study uses this deep learning model to automatically extract 1536 image features from a GMP layer.
(4) VGG16: VGG16 is a convolutional neural network model proposed by Simonyan et al. at ILSVRC-2014 [33], which includes 13 convolutional layers, five pooling layers, three fully connected (FC) layers, and finally the softmax layer. This model was used in the classification of the ImageNet dataset. There are a total of 1000 classes, as shown in Figure 1. This study uses this deep learning model to automatically extract 512 image features from a GMP layer automatically.
(5) Gerd-VGGNet: Taking VGG16 as the feature extractor, the classification architecture was designed and fine-tuned for the classification of GERD [20]. In the classification head composed with a GMP layer, a fully connected layer of 256, 128, 64, and 32 nodes and 4 batch normalization layers were added in sequence. Finally, a softmax layer was introduced. The detailed architecture is shown in Figure 1. This study uses this deep learning model to automatically extract 512, 256, 128, 64, and 32 features from the GMP layer and 4 different FC layers, respectively, as shown in Figure 1.

### 2.3. Random Forest Classifier

The random forest algorithm is a classifier that contains multiple decision trees, proposed by Breiman [34]. An ensemble learning technique uses the bagging method and random feature sampling. The random forest algorithm uses replacement sampling to generate multiple training sets, randomly selects independent variables, and generates multiple decision trees. Finally, the classification results of multiple trees are combined using majority voting.

Compared with the decision tree, the random forest algorithm is based on the ensemble learning technique and uses the majority decision to improve model performance. In addition, it is made up of multiple different trees, each of which is independent. Therefore, a random forest model is less likely to overfit and have high generalization, high accuracy for most datasets, and fast training speed.

Scikit-learn (Sklearn) [35] is a very robust and useful machine learning library in Python. After completing image feature extraction, this study compares 11 Sklearn machine learning classifiers and finally selects the random forest algorithm as the best classification model. The optimization of the configuration of a random forest classifier that includes the number of estimators, maximum depth, random state, and maximum features in this study.

### 2.4. Performance Evaluation

This study is a 3-class classification task rather than a binary classification task, and all experiments in this paper applied the accuracy value (ACC) and the Kappa value as model performance evaluation indicators to compare the differences in multiclass classification performance. The higher the ACC and Kappa values, the better the model’s classification performance. The detailed calculation formula is as follows:

ACC: For a given test set, the proportion of the total number of samples that the classifier correctly classified.

(1) $$\mathrm{ACC}=\frac{TP+TN}{TP+TN+FP+FN}$$

Kappa: This indicator is used to analyze the consistency of the classification model for each prediction of categories in the multiclass classification task. $P_{0}$ is the observed probability or the proportion of agreement and $P_{c}$ is the expected probability.

(2) $$\mathrm{Kappa}=\frac{P_{0}-P_{c}}{1-P_{c}}$$

### 2.5. Cross Validation

In this study, a 10-fold cross-validation is used for training and validation of the classification model. The development set was randomly and equally divided into ten subsets. A subset was used as the validation set, and the remaining nine subsets were used as the training set. In each training process, 608 images were used as the training set and the remaining 63 images were used as the validation set. This was repeated ten times until each subset was used as the validation set, and then the average of the classification results of 10 experiments was calculated as the training and validation results. Lastly, the ten trained classification models were used to evaluate the performance of the test set.

### 2.6. Proposed Framework

**Figure 2:**
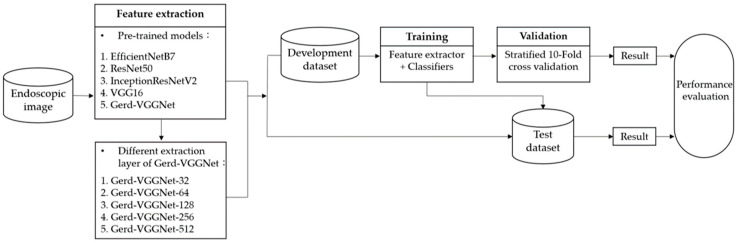
Study flowchart.

**Figure 3:**
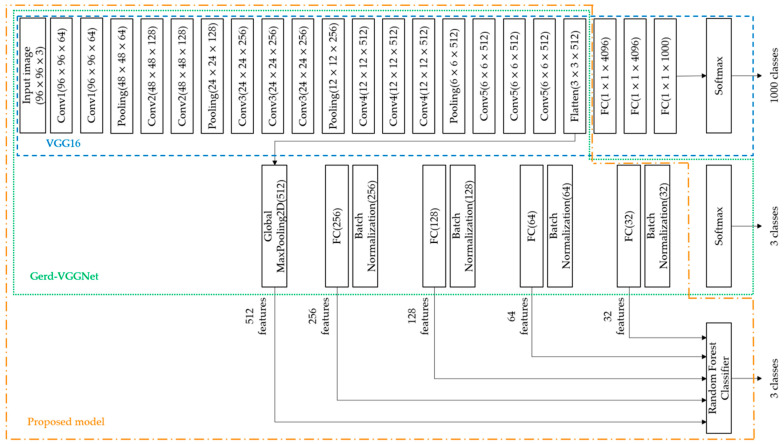
Proposed model architecture.

This study uses NBI images of gastroesophageal endoscopy to develop an improved classification model of GERD. The overall study flowchart is shown in Figure 2 and the detailed architecture proposed in this study is demonstrated in Figure 3. First, the development and test sets apply different pre-trained models for automatic image feature extraction, including EfficientNetB7, ResNet50, InceptionResNetV2, VGG16, and Gerd-VGGNet. For the Gerd-VGGNet model, this study uses the output of five different layers (one GMP and four FC) to extract 32, 64, 128, 256, and 512 features, respectively, as shown in Figure 3. Then, the extracted feature sets from the development and test sets are used to compare the classification performance of different machine learning algorithms and perform a 10 fold cross-validation to find the best classification model for GERD.

In order to evaluate the performance of image feature extraction technology, machine learning algorithm, parameter optimization of random forest classifier, and image feature extraction layer, this paper conducts four experiments, as detailed in Section 3.1, Section 3.2, Section 3.3 and Section 3.4.

The architecture proposed in this study can be divided into three blocks, as shown in Figure 3. The first part is the VGG16 pre-trained model, the second part describes Gerd-VGGNet [20], and the third part applies the machine learning classifier. The overall architecture of the proposed model takes VGG16 as the backbone, fine-tunes Gerd-VGGNet for image feature extraction, and finally uses a random forest classifier for GERD classification.

## 3. Experimental Result

This paper uses the feature data set extracted from the NBI images of GERD. It is divided into a development set and a test set, and the number of data is 671 and 32, respectively [20], in which the development set uses Stratified K-Fold to perform 10-fold cross-validation to train and verify the classification model and build 10 classification models. Finally, the performance evaluation of the target data set is carried out and the relevant experimental results are described below.

### 3.1. Performance Comparison of Image Feature Extraction Techniques

In this section, we first discuss the difference in the classification performance of GERD NBI images by using the traditional deep learning models with transfer learning and the random forest model with feature extraction by deep learning pre-trained models. 

Four popular CNN architectures including EfficientNetB7, ResNet50, InceptionResNetV2 and VGG16 are used as a feature extractor by loading the pre-trained network that does not include the classification layers at the top. For the traditional deep learning models with transfer learning technique, a GMP layer and a FC layer with 3 nodes are added as the classification head. This network of the Gerd-VGGNet model is proposed by Wang et al. [20] as shown in Figure 3. The classification architecture of Gerd-InceptionResNetV2 is the same as that of Gerd-VGGNet, except that the InceptionResNetV2 pre-trained model is used as the backbone. It should be noticed that the number of image features is 2560, 2048, 1536, 512, and 512 using the feature extractors of EfficientNetB7, ResNet50, InceptionResNetV2, VGG16, and Gerd-VGGNet, respectively.

Using five pre-trained models for feature extraction on GERD NBI images, the first six columns in Table 1 list the classification results of different deep learning models with transfer learning, and the last five columns were the classification performance of the random forest classifier without parameter optimization on extracted feature sets from different pre-trained models.

**Figure 4:**
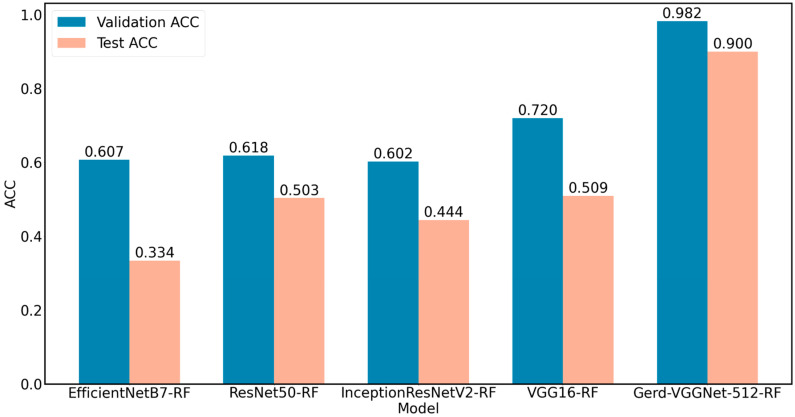
Comparison of Performance of five feature extractors.

The results in Table 1 show that among the six deep learning models with transfer learning, Gerd-VGGNet has the best classification performance. When using the random forest classifier with five deep learning pre-trained models, the classification performance of Gerd-VGGNet + RF is the best. In the study, many configurations using Gerd-VGGNet + RF have been tested. The 512 one works best. The Gerd-VGGNet + RF with the 512 configuration is named GerdNet-RF here. Furthermore, the results also reveal that the performance of using the random forest algorithm combined with GERD image feature set extracted by the pre-trained model is better than the traditional deep learning model with transfer learning. The results in Table 1 show that the best classification performance for image feature extraction is with the Gerd-VGGNet pre-trained model. The ACC and Kappa for the validation set are 0.982 ± 0.017 and 0.973 ± 0.026, respectively, while for the test set, they can increase to 0.900 ± 0.013 and 0.851 ± 0.019. Using Gerd-VGGNet as the image feature extractor for the GERD NBI images has the highest classification accuracy, as shown in Figure 4. It should be noted that the results of the GerdNet-RF model in Table 1 are without parameter optimization.

### 3.2. Performance Comparison of Machine Learning Algorithms

**Figure 5:**
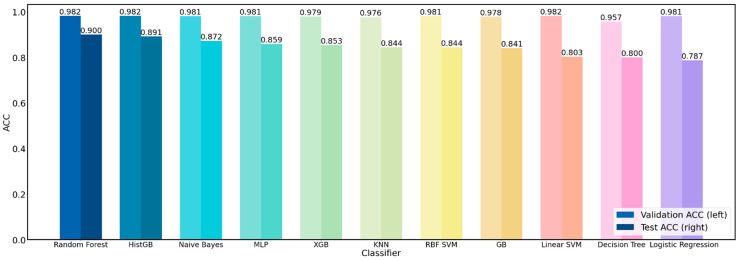
Comparison of performance of 11 classifiers.

Based on the 512 features extracted from the Gerd-VGGNet pre-trained model, this section compares the differences in the classification performance of 11 different machine learning algorithms [35] for endoscopic classification in GERD: Histogram-based gradient boosting classification tree (HistGB), extreme gradient boosting classification tree (XGB), gradient boosting classification tree (GB), random forest, K-nearest neighbors (KNN), linear support vector machine (Linear SVM), support vector machine with radial basis function kernel (RBF SVM), decision tree, Gaussian naive Bayes, logistic regression, and multilayer perceptron (MLP). All of these machine learning algorithms are employed in the Sklearn library, and their parameters were set as default values of the Sklearn library. The results of 11 different classifiers for GERD classification are compared in Table 2, demonstrating that the prediction performance of the use of the random forest classifier is significantly better than that of other machine learning algorithms. The ACC and Kappa are 0.982 ± 0.017 and 0.973 ± 0.026 for the validation set, and they can reach 0.900 ± 0.013 and 0.851 ± 0.019 for the test set. Therefore, for the GERD dataset, the random forest classifier (i.e., the GerdNet-RF model without parameter optimization) has the best classification performance, as shown in Figure 5. 

### 3.3. Performance Differences in Parameter Optimization of Random Forest Classifier

According to the above experiments, the best model is the random forest classifier. In this section, the grid search technique [35] is used to optimize the parameters of this GerdNet-RF model. Based on four important parameters of the random forest algorithm [34], including the number of estimators, maximum depth, random state, and maximum features, Table 3 lists the performance of 10 parameter optimization experiments for the GerdNet-RF classifier.

**Figure 6:**
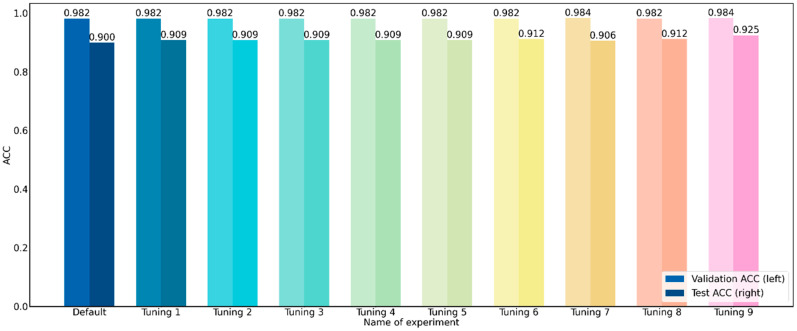
Comparison of performance of parameter optimization of GerdNet-RF classifier.

The results in Table 3 show that the performance is better when the number of estimators is equal to 200, the ACC can reach 0.982 ± 0.017 and 0.909 ± 0.009 for the validation and test sets. On the contrary, the number of estimators is set to 100, and the ACC of the test set is only 0.900 ± 0.017. When setting the maximum depth to 4, 8, and 16, it can be seen from Table 3 that when this parameter is set to 8 and 16, there is no significant difference. And the classification performance is better when the maximum depth is set to 4. The ACC can reach 0.912 ± 0.012 for the test set. Comparing the effect of max features on classification performance, when set to log2, the ACC of the validation set can be improved to 0.984 ± 0.018. By comparing the effect of the random state from 0 to 99 for dataset splitting and finding that the classification performance is the best when a random state is set to 81, the performances of the validation and test sets are both improved. The ACC can be improved to 0.984 ± 0.014 and 0.925 ± 0.021 for the validation and test sets. Finally, the optimal parameter combination for this GerdNet-RF model is the number of estimators equal to 200, the maximum depth equal to 4, the random state equal to 81, and the largest feature equal to log2, as shown in Figure 6. It should be noted that the default experiment in Table 3 is the GerdNet-RF classifier without parameter optimization, and the Tuning 9 experiment represents the GerdNet-RF model proposed in the study.

### 3.4. Performance Comparison of Image Feature Extraction Layers

**Figure 7:**
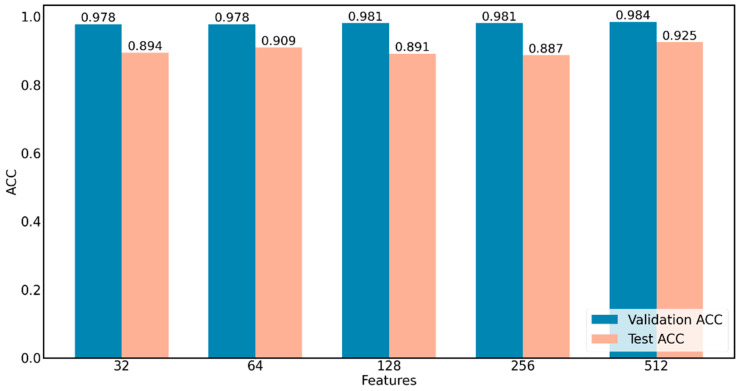
Comparison of Performance of five feature extraction layers of Gerd-VGGNet.

To compare the effect of different feature extraction layers of the Gerd-VGGNet pre-trained model on GERD classification performance, this study uses five different feature extraction layers, as shown in Figure 3. Table 4 lists the classification results when the number of extracted features is 32, 64, 128, 256, and 512. When the number of extracted features is small, the classification performance is relatively low. For example, while the number of extracted features is 32, the ACC of the test set can only reach 0.894 ± 0.021. When using 512 extracted features, the ACC of the test set can be increased to 0.925 ± 0.021, and the Kappa value can be improved to 0.888 ± 0.031, as shown in Figure 7. It should be noted that the experiment of 512 features in Table 4 corresponds to the GerdNet-RF model suggested in the article.

## 4. Discussion

### 4.1. Performance Comparison of Related Literature

Table 5 lists the classification performance comparison of AI models for the prediction of GERD with three recent publications. This study uses the same dataset as Wang et al. [20], with a total of 671 images for the 3-class classification task. The other two articles used 671 questionnaire data [18] and 147 image data [19] for binary classification tasks, respectively. 

Compared to Pace et al. [18] using ANN to predict whether a patient has GERD or not. The diagnosis and management of GERD-related complication is based mainly on endoscopic findings rather than symptom alone. The present study only needs to collect images without complicated questionnaire data collection and can predict the different endoscopic stages of gastroesophageal reflux disease. Such an AI model could be applied to the endoscopic electronic reporting system to help the endoscopist generate the endoscopic report in real time. 

The model suggested In this work can diagnose and grade GERD without any manual selection of the region of interest and achieves superior accuracy compared to the approach of Huang et al. [19]. Our proposed method is easier to apply in future work in the field, especially when manpower for image labeling is usually limited in clinical practice.

**Figure 8:**
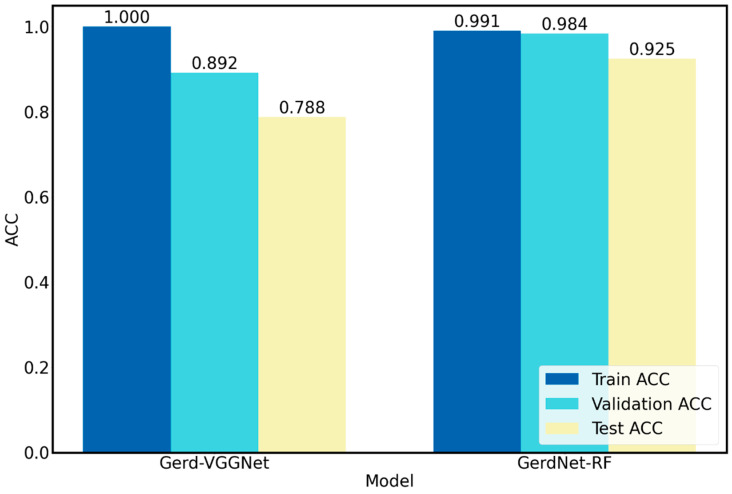
Comparison of performance of Gerd-VGGNet with proposed GerdNet-RF.

This study combines deep learning and machine learning approaches to produce an enhanced classification model in comparison to Wang et al. [20], who used deep learning with data augmentation to build the Gerd-VGGNet model. The classification results show a better performance improvement. The overall ACC has increased from 98.9% ± 0.5% to 99.0% ± 0.1%, and the test ACC has also improved from 78.8% ± 8.5% to 92.5% ± 2.1%, as shown in Figure 8. The results of Figure 8 clearly show that the Gerd-VGGNet [20] model has an overfitting problem. A superior GERD computer-aided diagnosis model for clinical practice applications is the GerdNet-RF model proposed in this work, which achieves very good generalization performance, and all training, validation, and test ACCs are very close.

### 4.2. Image Automatic Classification and Interpretation

To compare the differences between the Gerd-VGGNet model presented in Wang et al. [20] and the GerdNet-RF model proposed in this study in the predicted cases, Table 6 shows the examples of endoscopic images and LA classification that both models can correctly predict for the three classes. Table 7 lists the three endoscopic images and LA grading that the two models predict incorrectly, where the incorrect grading is marked in red. For example, in the first picture, both models misjudge A–B grades as C–D grades, and the second and third images are images that Gerd-VGGNet predict incorrectly.

## 5. Conclusions

This study proposes a two-stage process that integrates deep learning and machine learning techniques to perform the three-class endoscopic classification in GERD. The experimental results confirm that the model using Gerd-VGGNet as the image feature extractor and the random forest classifier with parameter optimization has the best performance, and the accuracies on the training set, the validation set, and the test set can reach 0.991, 0.984, and 0.925, respectively. Compared to the Gerd-VGGNet model of Wang et al. [20], the GerdNet-RF model proposed in this work can achieve higher classification performance, with an average accuracy improvement rate of 13.7% in the test set. The accuracy of the proposed model also outperforms previous methods proposed by various researchers in the past.

This artificial intelligence model proposed in this study can be applied to an endoscope electronic reporting system to help endoscopists generate endoscopic reports in real time. Especially when the manpower for image labeling in clinical practice is usually limited, our proposed method makes it easier to achieve a computer-aided application in this field.

## Figures and Tables

**Table 1:** Performance evaluation of five feature extractors.

Model	Features	Training		Validation		Test	
		Accuracy	Kappa	Accuracy	Kappa	Accuracy	Kappa
EfficientNetB7	-	0.348 ± 0.027	0.000 ± 0.000	0.349 ± 0.026	0.000 ± 0.000	0.347 ± 0.082	−0.016 ± 0.123
ResNet50	-	0.480 ± 0.015	0.208 ± 0.024	0.478 ± 0.070	0.206 ± 0.108	0.369 ± 0.027	0.016 ± 0.041
InceptionResNetV2	-	0.789 ± 0.016	0.682 ± 0.024	0.687 ± 0.036	0.530 ± 0.054	0.556 ± 0.056	0.329 ± 0.083
VGG16	-	0.681 ± 0.013	0.520 ± 0.019	0.653 ± 0.042	0.479 ± 0.064	0.503 ± 0.053	0.254 ± 0.080
Gerd- InceptionResNetV2	-	0.999 ± 0.001	0.999 ± 0.001	0.879 ± 0.022	0.818 ± 0.033	0.684 ± 0.051	0.520 ± 0.078
Gerd-VGGNet	-	0.999 ± 0.001	0.999 ± 0.001	0.903 ± 0.022	0.854 ± 0.034	0.737 ± 0.037	0.603 ± 0.057
EfficientNetB7-RF	2560	1.000 ± 0.000	1.000 ± 0.000	0.607 ± 0.045	0.407 ± 0.068	0.334 ± 0.037	−0.026 ± 0.057
ResNet50-RF	2048	1.000 ± 0.000	1.000 ± 0.000	0.618 ± 0.076	0.422 ± 0.114	0.503 ± 0.033	0.235 ± 0.052
InceptionResNetV2-RF	1536	1.000 ± 0.000	1.000 ± 0.000	0.602 ± 0.019	0.397 ± 0.028	0.444 ± 0.057	0.164 ± 0.085
VGG16-RF	512	1.000 ± 0.000	1.000 ± 0.000	0.720 ± 0.049	0.577 ± 0.074	0.509 ± 0.061	0.255 ± 0.093
GerdNet-RF	512	1.000 ± 0.000	1.000 ± 0.000	0.982 ± 0.017	0.973 ± 0.026	0.900 ± 0.013	0.851 ± 0.019

**Table 2:** Performance evaluation of 11 classifiers based on 512 features of Gerd-VGGNet feature extractor.

Classifier	Training		Validation		Test	
	Accuracy	Kappa	Accuracy	Kappa	Accuracy	Kappa
Random Forest	1.000 ± 0.000	1.000 ± 0.000	0.982 ± 0.017	0.973 ± 0.026	0.900 ± 0.013	0.851 ± 0.019
HistGB	1.000 ± 0.000	1.000 ± 0.000	0.982 ± 0.013	0.973 ± 0.020	0.891 ± 0.021	0.836 ± 0.031
Naive Bayes	0.984 ± 0.002	0.976 ± 0.003	0.981 ± 0.013	0.971 ± 0.020	0.872 ± 0.009	0.809 ± 0.014
MLP	0.982 ± 0.003	0.973 ± 0.004	0.981 ± 0.018	0.971 ± 0.027	0.859 ± 0.047	0.790 ± 0.070
XGB	1.000 ± 0.000	1.000 ± 0.000	0.979 ± 0.017	0.969 ± 0.025	0.853 ± 0.034	0.781 ± 0.051
KNN	0.982 ± 0.003	0.973 ± 0.004	0.976 ± 0.015	0.964 ± 0.023	0.844 ± 0.000	0.765 ± 0.001
RBF SVM	0.982 ± 0.002	0.974 ± 0.003	0.981 ± 0.018	0.971 ± 0.027	0.844 ± 0.014	0.766 ± 0.021
GB	1.000 ± 0.000	1.000 ± 0.000	0.978 ± 0.017	0.966 ± 0.025	0.841 ± 0.033	0.762 ± 0.048
Linear SVM	0.997 ± 0.001	0.995 ± 0.002	0.982 ± 0.017	0.973 ± 0.026	0.803 ± 0.014	0.705 ± 0.021
Decision Tree	1.000 ± 0.000	1.000 ± 0.000	0.957 ± 0.028	0.935 ± 0.042	0.800 ± 0.051	0.701 ± 0.075
Logistic Regression	0.994 ± 0.001	0.991 ± 0.002	0.981 ± 0.022	0.971 ± 0.033	0.787 ± 0.019	0.682 ± 0.028

**Table 3:** Performance evaluation of parameter optimization of GerdNet-RF classifier.

Name of Experiment	Number of Estimators	Max Depth	Random State	Max Features	Training		Validation		Test	
					Accuracy	Kappa	Accuracy	Kappa	Accuracy	Kappa
Default	100	None	1	sqrt	1.000 ± 0.000	1.000 ± 0.000	0.982 ± 0.017	0.973 ± 0.026	0.900 ± 0.013	0.851 ± 0.019
Tuning 1	200	None	1	sqrt	1.000 ± 0.000	1.000 ± 0.000	0.982 ± 0.017	0.973 ± 0.026	0.909 ± 0.009	0.865 ± 0.014
Tuning 2	300	None	1	sqrt	1.000 ± 0.000	1.000 ± 0.000	0.982 ± 0.017	0.973 ± 0.026	0.909 ± 0.009	0.865 ± 0.014
Tuning 3	400	None	1	sqrt	1.000 ± 0.000	1.000 ± 0.000	0.982 ± 0.017	0.973 ± 0.026	0.909 ± 0.009	0.865 ± 0.014
Tuning 4	200	16	1	sqrt	1.000 ± 0.000	1.000 ± 0.000	0.982 ± 0.017	0.973 ± 0.026	0.909 ± 0.009	0.865 ± 0.014
Tuning 5	200	8	1	sqrt	1.000 ± 0.000	1.000 ± 0.000	0.982 ± 0.017	0.973 ± 0.026	0.909 ± 0.009	0.865 ± 0.014
Tuning 6	200	4	1	sqrt	0.992 ± 0.002	0.988 ± 0.003	0.982 ± 0.017	0.973 ± 0.026	0.912 ± 0.012	0.869 ± 0.019
Tuning 7	200	4	1	log2	0.990 ± 0.002	0.985 ± 0.003	0.984 ± 0.018	0.975 ± 0.027	0.906 ± 0.020	0.860 ± 0.030
Tuning 8	200	4	81	sqrt	0.992 ± 0.002	0.988 ± 0.003	0.982 ± 0.017	0.973 ± 0.026	0.912 ± 0.012	0.869 ± 0.019
Tuning 9	200	4	81	log2	0.991 ± 0.001	0.986 ± 0.002	0.984 ± 0.014	0.975 ± 0.021	0.925 ± 0.021	0.888 ± 0.031

**Table 4:** Performance evaluation of five feature extraction layers of Gerd-VGGNet.

Features	Training		Validation		Test	
	Accuracy	Kappa	Accuracy	Kappa	Accuracy	Kappa
32	0.994 ± 0.002	0.990 ± 0.002	0.978 ± 0.012	0.966 ± 0.018	0.894 ± 0.021	0.841 ± 0.031
64	0.993 ± 0.001	0.989 ± 0.001	0.978 ± 0.012	0.966 ± 0.018	0.909 ± 0.026	0.864 ± 0.039
128	0.993 ± 0.001	0.990 ± 0.002	0.981 ± 0.012	0.971 ± 0.018	0.891 ± 0.029	0.835 ± 0.044
256	0.993 ± 0.001	0.989 ± 0.002	0.981 ± 0.012	0.971 ± 0.018	0.887 ± 0.032	0.831 ± 0.048
512	0.991 ± 0.001	0.986 ± 0.002	0.984 ± 0.014	0.975 ± 0.021	0.925 ± 0.021	0.888 ± 0.031

**Table 5:** Performance comparison of different AI systems for classification of GERD.

Task	Algorithm	Data Used	Evaluation Method	Overall Accuracy	Test Accuracy
Binary classification	Machine learning (ANN) [18]	QUID ^1^ questionnaire (577 GERD ^2^ patients, 94 normal cases)	hold-out	99.2%	NA
Binary classification	Machine learning (HHDF-SVM) [19]	147 RGB images (39 GERD patients, 108 normal cases)	10-fold cross-validation	93.2%	NA
3-class classification	Deep learning (Gerd-VGGNet) [20]	671 NBI ^3^ images (GERD A–B: GERD C–D: normal EC-J = 244:229:198)	10-fold cross-validation	98.9% ± 0.5%	78.8% ± 8.5%
3-class classification	Deep learning + Machine learning (proposed GerdNet-RF)	671 NBI ^3^ images (GERD A–B: GERD C–D: normal EC-J = 244:229:198)	10-fold cross-validation	99.0% ± 0.1%	92.5% ± 2.1%

^1^ QUestionario Italiano Diagnostico = QUID; ^2^ Gastroesophageal reflux disease = GERD; ^3^ Narrow-band imaging = NBI.

**Table 6:** Three types of examples where both models predict correctly.

Image	GERD Endoscopy Grading	Gerd-VGGNet	GerdNet-RF
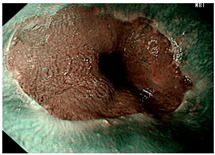	LA grade A–B	LA grade A–B	LA grade A–B
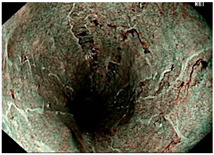	LA grade C–D	LA grade C–D	LA grade C–D
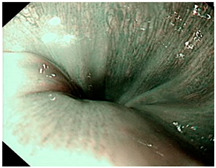	LA grade normal	LA grade normal	LA grade normal

**Table 7:** Three types of examples where two models predict incorrectly.

Image	GERD Endoscopy Grading	Gerd-VGGNet	GerdNet-RF
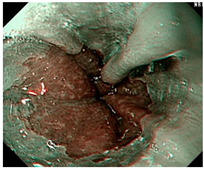	LA grade A–B	LA grade C–D	LA grade C–D
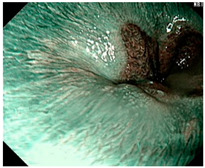	LA grade A–B	LA grade C–D	LA grade A–B
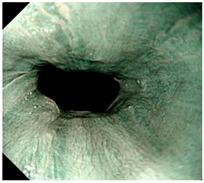	LA grade normal	LA grade C–D	LA grade normal

## Data Availability

All the data of images and analysis process were kept at the lab of M.-H.T.

## Abbreviations

**Abbreviation**	**Name**
GERD	gastroesophageal reflux disease
LA	Los Angeles
AI	artificial intelligence
QUID	QUEstionario Italiano Diagnostico
ANN	artificial neural network
RF	Random Forest
HHDF-SVM	hierarchical heterogeneous descriptor fusion support vector machine
NBI	Narrow Band Imaging
FC	Fully Connected
GMP	Global Maxinum Pooling
Sklearn	Scikit-learn
ACC	Accuracy
HistGB	Histogram-based Gradient Boosting
XGB	eXtreme Gradient Boosting
KNN	K-nearest Neighbors
Linear SVM	Linear Support Vector Machine
RBF SVM	Support Vector Machine with Radial Basis Function kernel
MLP	Multi-layer Perceptron
